# Cut-off Scores of a Brief Neuropsychological Battery (NBACE) for Spanish Individual Adults Older than 44 Years Old

**DOI:** 10.1371/journal.pone.0076436

**Published:** 2013-10-16

**Authors:** Montserrat Alegret, Ana Espinosa, Sergi Valero, Georgina Vinyes-Junqué, Agustín Ruiz, Isabel Hernández, Maitee Rosende-Roca, Ana Mauleón, James T. Becker, Lluís Tárraga, Mercè Boada

**Affiliations:** 1 Memory Clinic, Fundació ACE, Institut Català de Neurociències Aplicades, Barcelona, Spain; 2 Department of Psychiatry, Hospital Universitari Vall d’Hebron, CIBERSAM, Universitat Autònoma de Barcelona, Barcelona, Spain; 3 Department of Psychiatry, University of Pittsburgh School of Medicine, Pittsburgh, Pennsylvania, United States of America; 4 Department of Neurology, University of Pittsburgh School of Medicine, Pittsburgh, Pennsylvania, United States of America; 5 Department of Psychology, University of Pittsburgh School of Medicine, Pittsburgh, Pennsylvania, United States of America; 6 Hospital Universitari Vall d’Hebron -Institut de Recerca, Universitat Autònoma de Barcelona (VHIR-UAB), Barcelona, Spain; University of São Paulo, Brazil

## Abstract

The neuropsychological battery used in Fundació ACE (NBACE) is a relatively brief, and easy to administer, test battery that was designed to detect cognitive impairment in the adulthood. The NBACE includes measures of cognitive information processing speed, orientation, attention, verbal learning and memory, language, visuoperception, praxis and executive functions. The aim of the present study was to establish the cut-off scores for impairment for different levels of age and education that could be useful in the cognitive assessment of Spanish subjects who are at risk for cognitive impairment, especially dementia. Data from 1018 patients with a mild dementia syndrome, and 512 cognitively healthy subjects, older than 44 years, from the Memory Clinic of *Fundació ACE* (Barcelona, Spain) were analyzed. In the whole sample, cut-off scores and sensitivity/specificity values were calculated for six conditions after combining 3 age ranges (44 to 64; 65 to 74; and older than 74 years old) by 2 educational levels (until Elementary school; and more than Elementary school). Moreover, general cut-offs are reported for Catalan and Spanish speakers. The results showed that most of NBACE tests reached good sensitivity and specificity values, except for Ideomotor praxis, Repetition and Verbal Comprehension tests, which had a ceiling effect. Word List Learning from the Wechsler Memory Scale-III and Semantic Verbal Fluency were the most useful tests to discriminate between cognitively healthy and demented subjects. The NBACE has been shown to be a useful tool able to detect cognitive impairment, especially dementia, in older than 44 years Spanish persons.

## Introduction

Neuropsychological assessment is critical for the diagnosis of clinical dementia syndromes or other forms of cognitive impairment [1], [2]. Subtle cognitive deficits generally cannot be detected by the screening tools frequently used by primary care physicians (e.g., Mini-Mental State Examination, MMSE) [3]. So, there is a need for brief neuropsychological batteries, covering a wide range of cognitive functions, able to detect subtle deficits in clinical settings.

In the clinical practice, raw neuropsychological test scores are adjusted for socio-demographic factors in order to facilitate an accurate interpretation of test performance. The scores can be adjusted in different ways, such as using normative data covering a range of tests [4], [5], [6], [7] or normative data from specific populations, such as the community-dwelling elders [8], [9].

In Spain, tests have been usually translated from English versions and adapted using foreign population normative data. To aid this process, projects like the Spanish Multicenter Normative Studies (NEURONORMA) have been adapting English language tests for the Spanish-speaking population [10], [11], [12], [13], [14]. A few neuropsychological screening batteries, adapted for Spanish-speaking individuals, have also been adapted to include age and education adjusted normative values [15], [16], [17], [18], [19]. All of these tests were standardized by administering them to a representative sample of cognitively healthy individuals to establish normative scores.

Neuropsychologists generally use a threshold of 1.5 standard deviation units below the performance mean of healthy population as an indication of impaired function [20], [21]. However, this strategy has the limitation that the classification of “abnormal” depends on the characteristics of the normative sample. Furthermore, the cut-off scores calculated using this strategy have a mathematical meaning that sometimes does not meet the clinical need. This psychometric approach does not necessarily provide an empirical estimation of the relevant cut-off value for abnormal performance. To establish the limits between preserved and impaired function, it is important to include patients with a known pathological condition (e.g. patients with dementia), which allows the calculation of sensitivity (probability to detect a dementia case) and specificity (probability to detect a non-dementia case) values for a test score.

Some international studies report cut-off scores of cognitive tests, including screening tools, such as the MMSE or the Montreal Cognitive Assessment (MoCA) [22], [23], [24], and complete neuropsychological test batteries (CERAD: [25]; RAPID: [26]). In Spain, cut-off scores are available for some screening tools (MMSE: [27]; 7-Minute Screening: [28]) and memory tests (Memory Impairment Screen: [29]; Test of Memory or T@M: [30]), but not for more complete neuropsychological batteries.

The NBACE is a brief neuropsychological battery developed by Fundació ACE for use in the Memory Clinic as a component of the diagnostic process of cognitive impairment and dementia [19]. It assesses the cognitive functions relevant to the diagnosis of neurodegenerative disorders in a reasonable amount of time, approximately 45 minutes. NBACE includes measures of processing speed, attention, verbal learning and memory, language, visuospatial ability, visuoperception, praxis and executive functions. Our group has recently reported the standardized NBACE scores and percentile ranks in cognitively healthy Spanish persons older than 49 years, for different levels of age and education [19], [31]. The aim of the present study was to establish the cut-off scores for impairment for different levels of age and education that could be useful in the cognitive assessment of Spanish subjects who are at risk for cognitive impairment, especially dementia.

## Methods

### Participants

*Fundació ACE, Institut Català de Neurociències Aplicades,* is a non-profit Alzheimer’s center that provides diagnostic, treatment and patient management services to the Catalan Public Health Service (*Xarxa Hospitalària d’Utilització Pública (XHUP)*). The patients are usually referred to the Memory Clinic of *Fundació ACE* by primary care physicians or medical specialists because the patients, their family, or their physician felt that they could have a memory problem [31].

The data included for the purpose of the present study were drawn from individuals older than 44 who were examined at *Fundació ACE* between September 2008 and January 2013. To establish cut-off scores, data from 1018 patients with a “Mild Dementia Syndrome” (MDS) and 512 cognitively healthy subjects (HS) were analysed (Table 1).

**Table 1:** Characteristics of participants.

	HS	MDS	Effect Size^1^
n	512	1018	
Age	66.2	79.1	1.46**
Education:			0.27**
≤Elementary school (%)	45.5	73.1	
>Elementary school (%)	54.5	26.9	
Gender (% Male)	36.9	34.3	0.026
MMSE	28.7	23.2	3.02**

HS: healthy subjects; MDS: mild dementia syndrome; MMSE: Mini-Mental State Examination.

1 Cohens’ d for t-tests in age and MMSE, and ^2^Phi for Chi square in education and gender.

* p<0.05,

** p<0.005.

General inclusion criteria were predefined as follows: Subjects older than 44 years old, without severe auditory or visual abnormalities that could affect performance on neuropsychological tests. With regard to MDS group, inclusion criteria were: A diagnosis of MDS, with a mild severity of dementia determined by a total Clinical Dementia Rating (CDR; [32]) of 1 and a Mini-Mental State Examination (MMSE; [27]) higher than 19. With regard to HS group, inclusion criteria were: A diagnosis of “without cognitive impairment”, a CDR of zero, preserved scores on the Memory test of the 7 Minute Screen test [33] (≥12), on the MMSE ((≥24) and on the Spanish version of the Clock Test [34] (≥5), and they had no evidence of functional impairment due to cognitive decline, with a score lower than 4 on the Blessed Dementia Rating Scale (BDRS) [35], [36] and a score lower than 17 in the Rapid Disability Rating Scale-2 (RDRS-2) [36], [37]).

Those subjects younger than 45 years old, with analphabetism or with severe auditory or visual abnormalities that could interfere on neuropsychological test performances were excluded from the study.

Written informed consent was obtained from all participants, and their caregivers in case of dementia, prior to any evaluation. The study was approved by the Fundació ACE Research Ethics Committee.

### Measures

All of the participants received an extensive clinical evaluation including a neurological history and examination, a semi-structured psychosocial interview conducted by a social worker and the NBACE assessment.

The neurologist performed a complete neurological exam, and administered the Tinetti balance and gait scale [38], the Hachinski Ischemia Scale [39], the MMSE [27], the Memory test of the 7 Minute Screen test [28], the Spanish Clock Test [34], the short form of the Neuropsychiatric Inventory-Questionnaire (NPI-Q) [40], the BDRS [35] and RDRS-2 [37].

All of the neuropsychological testing was carried out in the Diagnostic Unit of *Fundació ACE* by one of the three neuropsychologists (MA, AE, and GVJ), and it was completed in the patient’s language of choice, which could be, or not, the first language learned (the 54.8% were assessed in Catalan and the remainder in Spanish). As described elsewhere [19], the NBACE includes the following tests: Temporal, Spatial and Personal Orientation; Digit Span (forward and backwards), Block Design (abbreviated so that items 6 to 9 were scored only for accuracy (1 point) without a time bonus) and Similarities (abbreviated to the first 10 items) subtests extracted and adapted from the Wechsler Adult Intelligence Scale-Third Edition (WAIS-III); The Word List Learning test from the Wechsler Memory Scale-Third Edition (WMS-III) (without using the interference list); Repetition (2 words and 2 sentences);Verbal comprehension (to correctly execute 2 simple, 2 semi-complex and 2 complex commands extracted from the ADAS-Cog and the Barcelona test battery); an abbreviated 15-item Boston Naming Test (15-BNT); the Poppelreuter test; Luria’s Clock test; the Automatic Inhibition subtest of the Syndrom Kurtz Test (SKT); Phonetic Verbal Fluency (words beginning with ‘P’ in one minute); Semantic Verbal Fluency (‘animals’ in one minute); 4 item Imitation praxis; 4 item Ideomotor commands; and the 15-Objects Test (15-OT). Moreover, global scores were calculated for orientation (5 Temporal+5 Spatial+5 Personal) and praxis (4 Imitation praxis+4 Ideomotor praxis+4 Block Design). In Table 2, score ranges are detailed.

**Table 2:** NBACE score descriptives and area under the curve values for the whole sample.

TEST	Range	HS	MDS	AUC	95% CI
		Mean (SD)	Mean (SD)		
Global orientation	0–15	14.9 (0.4)	12.3 (2.0)	0.89	0.88–0.91
Verbal Learning WMS-III#	0–48	28.8 (5.7)	13.6 (4.7)	0.98	0.97–0.98
Delayed recall WMS-III	0–12	7.0 (2.5)	0.7 (1.3)	0.98	0.98–0.99
Forgetting WMS-III (%)	0–100	27.6 (19.5)	85.5 (26.8)	0.94	0.92–0.95
Recognition Memory WMS-III	0–24	22.6 (1.6)	16.9 (3.2)	0.94	0.93–0.95
Digit Span Forward WAIS-III	0–16	5.4 (1.1)	4.7 (0.9)	0.69	0.65–0.71
Digit Span Backwards WAIS-III	0–14	3.9 (1.0)	2.7 (0.9)	0.81	0.78–0.83
Block Design WAIS-III	0–4	3.9 (0.5)	2.1 (1.5)	0.84	0.82–0.86
Imitation praxis	0–4	3.8 (0.8)	2.4 (1.3)	0.82	0.80–0.84
Global praxis	0–12	11.6 (0.9)	8.4 (2.4)	0.90	0.89–0.92
Visual Naming (15-BNT)	0–15	14.6 (0.7)	11.5 (3.0)	0.86	0.84–0.88
Poppelreuter’s test (responses)	0–10	9.9 (0.4)	8.1 (1.9)	0.82	0.80–0.84
15-OT (responses)‡	0–15	13.6 (1.6)	9.4 (2.6)	0.84	0.91–0.95
Luria’s Clock test	0–4	3.5 (0.7)	1.9 (1.3)	0.84	0.83–0.86
Automatic inhibition SKT (s)	≥0	26.8 (7.7)	49.8 (21.3)	0.89	0.87–0.90
Automatic Inhibition SKT (error)	0–34	0.8 (1.6)	6.9 (6.7)	0.77	0.85–0.88
Phonetic verbal fluency	≥0	14.0 (4.4)	7.5 (4.1)	0.87	0.85–0.89
Semantic verbal fluency	≥0	18.7 (4.7)	8.9 (3.8)	0.95	0.94–0.96
Similarities WAIS-III	0–15	11.4 (2.0)	7.0 (2.9)	0.89	0.87–0.91

HS: healthy subjects; MDS: mild dementia syndrome; SD: standard deviation; AUC: area under the curve; CI: Confidence Interval; WMS-III: Wechsler Memory Scale, Third Edition; WAIS-III: Wechsler Adult Intelligence Scale, Third edition; 15-BNT: the abbreviated Boston Naming Test with 15 items; 15-OT: The 15-Objects test; SKT: Syndrom Kurtz Test; s: time in seconds;

# Verbal learning WMS-III = 1^st^+2^nd^+3^rd^+4^th^ trial scores.

‡ In a subsample of 246 HS and 271 MDS.

### Procedure

The preliminary diagnosis of each individual was initially made by the neurologist based only on the clinical history and exam, and not on the NBACE results. The initial classification was later reviewed by the study team, including neurologists, neuropsychologists and social workers, at a consensus diagnostic conference. The dementia diagnosis was mainly based on the impairment on the activities of daily living. Functionality was assessed by the BDRS [35] and the RDRS-2 [37], both of them validated in Spanish population [36]. A score higher than 4 on the BDRS, and a score higher than 16 on the RDRS-2, meant that the subject was dependent on their activities of daily living [36]. Once the dementia was diagnosed, NBACE performances profile contributed to specify the dementia type.

### Statistical Analysis

Statistical analysis was carried out using SPSS 20 (SPSS Inc., Chicago, IL). T-tests were used to compare age and MMSE scores between groups, and Chi-square for education and gender comparisons. A sample of 1018 patients with a MDS and 512 HS was used to calculate cut-off scores and sensitivity/specificity values for six conditions after combining 3 age ranges (44 to 64; 65 to 74; and older than 74 years) by 2 educational levels (through elementary school (≤8 years); and more than elementary school (>8 years)) (see Table 3). Multiple imputation methods were used to account for any missing test data, which was ∼0.3% of the total.

**Table 3:** Sample sizes of each of the 6 conditions (3 age ranges by 2 educational levels).

Educational level/Age	45–64		65–74		>74		
	HS	MDS	HS	MDS	HS	MDS	Total
≤Elementary school	81	33	80	106	72	605	*977*
>Elementary school	142	27	91	51	46	196	*553*
Total	*223*	*60*	*171*	*157*	*118*	*801*	*1530*

HS: healthy subjects; MDS: mild dementia syndrome.

For the whole sample, and for each language subgroup (Catalan and Spanish), cut-off points were established by calculating the sensitivity and specificity for each test score. Receiver operating characteristic (ROC) analysis was used to calculate the optimal cut-off value between MDS and HS groups. Moreover, to calculate the sensitivity and specificity of each cut-off score, an area under the curve (AUC) analysis was also carried out including its confidence interval 95%.

Our goal was to obtain an AUC greater than 0.75 for each test variable and for the most of 6 (age by education) conditions, sensitivity and specificity values were greater than 0.75. For those variables where sensitivity and specificity values were lower than 0.75, a unique cut-off score was calculated for the entire sample adjusting by age and educational level.

## Results

MDS patients were older and had a lower educational level than HS (See Table 1). For the purpose of the present study, it was not a problem, because cut-offs were reported after combining 3 age ranges (45–64, 65–74 and >74) by 2 educational levels (≤ Elementary school and>Elementary school) (Tables 4–12), and global cut-offs were adjusted by age and education (Table 13).

**Table 4:** Cut-off scores for 6 conditions (3 age ranges by 2 educational levels), including sensitivity (SE) and specificity (SP) values.

Test	Range	Educationallevel	Age		
			45–64	65–74	≥75
Verbal LearningWMS-III#	0–48	≤Elementary	<24	<22	<20
		School	SE = 91	SE = 95	SE = 91
			SP = 89	SP = 90	SP = 88
		>Elementary	<24	<23	<21
		School	SE = 96	SE = 92	SE = 90
			SP = 94	SP = 92	SP = 80

WMS-III: Wechsler Memory Scale, Third Edition;

# Verbal learning WMS-III = 1^st^+2^nd^+3^rd^+4^th^ trial scores.

**Table 5:** Cut-off scores for 6 conditions (3 age ranges by 2 educational levels), including sensitivity (SE) and specificity (SP) values.

Test	Range	Educationallevel	Age		
			45–64	65–74	≥75
Delayed RecallWMS-III	0–12	≤Elementary	<5	<4	<3
		School	SE = 94	SE = 90	SE = 92
			SP = 91	SP = 94	SP = 99
		>Elementary	<5	<5	<3
		School	SE = 93	SE = 92	SE = 93
			SP = 92	SP = 91	SP = 85

WMS-III: Wechsler Memory Scale, Third Edition.

**Table 6:** Cut-off scores for 6 conditions (3 age ranges by 2 educational levels), including sensitivity (SE) and specificity (SP) values.

Test	Range	Educationallevel	Age		
			45–64	65–74	≥75
ForgettingWMS-III (%)	0–100	≤Elementaryschool	>37	>40	>55
			SE = 88	SE = 83	SE = 90
			SP = 84	SP = 79	SP = 89
		>Elementaryschool	>33	>41	>60
			SE = 78	SE = 76	SE = 87
			SP = 80	SP = 81	SP = 76

WMS-III: Wechsler Memory Scale, Third Edition.

**Table 7:** Cut-off scores for 6 conditions (3 age ranges by 2 educational levels), including sensitivity (SE) and specificity (SP) values.

Test	Range	Educationallevel	Age		
			45–64	65–74	≥75
Recognition memoryWMS-III	0–24	≤Elementary	<22	<22	<21
		school	SE = 79	SE = 91	SE = 86
			SP = 84	SP = 84	SP = 82
		>Elementary	<22	<22	<21
		school	SE = 85	SE = 90	SE = 90
			SP = 85	SP = 82	SP = 72

WMS-III: Wechsler Memory Scale, Third Edition.

**Table 8:** Cut-off scores for 6 conditions (3 age ranges by 2 educational levels), including sensitivity (SE) and specificity (SP) values.

Test	Range	Educationallevel	Age		
			45–64	65–74	≥75
Visual Naming(15-BNT)	0–12	≤Elementary school	<15	<15	<14
			SE = 82	SE = 83	SE = 75
			SP = 73	SP = 63	SP = 88
		>Elementary school	<15	<15	<15
			SE = 70	SE = 76	SE = 75
			SP = 84	SP = 87	SP = 80

15-BNT: the abbreviated Boston Naming Test with 15 items.

**Table 9:** Cut-off scores for 6 conditions (3 age ranges by 2 educational levels), including sensitivity (SE) and specificity (SP) values.

Test	Range	Educationallevel	Age		
			45–64	65–74	≥75
PhoneticVerbalFluency	>0	≤Elementaryschool	<11	<11	<10
			SE = 76	SE = 84	SE = 76
			SP = 66	SP = 70	SP = 78
		>Elementaryschool	<12	<12	<13
			SE = 78	SE = 80	SE = 78
			SP = 77	SP = 82	SP = 74

**Table 10:** Cut-off scores for 6 conditions (3 age ranges by 2 educational levels), including sensitivity (SE) and specificity (SP) values.

Test	Range	Educationallevel	Age		
			45–64	65–74	≥75
Semantic VerbalFluency	>0	≤Elementaryschool	<14	<14	<13
			SE = 82	SE = 84	SE = 85
			SP = 83	SP = 84	SP = 83
		>Elementaryschool	<16	<15	<13
			SE = 93	SE = 98	SE = 81
			SP = 85	SP = 96	SP = 87

**Table 11:** Cut-off scores for 6 conditions (3 age ranges by 2 educational levels), including sensitivity (SE) and specificity (SP) values.

Test	Range	Educationallevel	Age		
			45–64	65–74	≥75
SimilaritiesWAIS-III	0–15	≤Elementary school	<10	<10	<10
			SE = 79	SE = 79	SE = 85
			SP = 77	SP = 66	SP = 74
		>Elementary school	<12	<11	<11
			SE = 78	SE = 72	SE = 78
			SP = 67	SP = 83	SP = 65

WAIS-III: Wechsler Adult Intelligence Scale, Third edition.

**Table 12:** Cut-off scores for 6 conditions (3 age ranges by 2 educational levels), including sensitivity (SE) and specificity (SP) values.

Test	Range	Educational level	Age		
			45–64	65–74	≥75
SKT Inhibitiontime	0–24	≤Elementary school	>31	>34	>35
			SE = 85	SE = 75	SE = 78
			SP = 80	SP = 76	SP = 79
		>Elementary school	>26	>27	>27
			SE = 74	SE = 86	SE = 77
			SP = 75	SP = 80	SP = 70

SKT: Syndrom Kurtz Test.

**Table 13:** Global cut-off scores.

TEST	Cut-off	SE	SP
Global orientation	<15	88.2	84.1
Digit Span Forward WAIS-III	<6	85.0	73.0
Digit Span Backwards WAIS-III	<4	87.9	76.5
Block Design WAIS-III	<4	86.1	86.3
Imitation praxis	<4	87.0	82.0
Global praxis	<11	92.2	84.2
Poppelreuter’s test (responses)	<10	86.1	83.2
Luria’s Clock test	<3	86.9	83.0
15-OT (responses)	<13	90.0	90.2
Automatic Inhibition SKT (error)	>2	86.5	83.6

SE: sensitivity; SP: specificity; WAIS-III: Wechsler Adult Intelligence Scale, Third edition; 15-OT: The 15-Objects test; SKT: Syndrom Kurtz Test.

The MDS patients met criteria for dementia [41], specifically 576 (56.6%) met criteria for Alzheimer’s disease (AD) [41], 143 (14.0%) vascular dementia (VD; NINCS-AIREN criteria: [42]), 75 (7.4%) mixed dementia (AD with cerebrovascular disease), 99 (9.7%) frontotemporal dementia (FTD; [43], [44]), 70 (6.9%) dementia with parkinsonism (PKS; mainly Parkinson’s Disease Dementia: [45] and Lewy Body Dementia: [46]), and 55 (5.4%) other dementia syndromes.

The AUC, means and SD of the NBACE test variables are detailed in Table 2. When the AUC was greater than 0.75, the cut-offs were reported after identifying the cut-off score that yielded approximately equal sensitivity and specificity values. As shown in Table 2, the measures that best discriminated between MDS and HS groups were the Word List Verbal Learning and Semantic Verbal Fluency tests.

Tables 4–12 provide the age and education adjusted cut-off scores for verbal learning and long-term memory (including recognition memory and forgetting), language and executive functioning. However, Global orientation, Digit span Forward and Backwards, Block Design, Imitation and Global praxis, Poppelreuter’s test, Luria’s Clock test, 15-OT and Automatic Inhibition SKT subtest (number of errors) showed sensitivity and specificity values for several conditions lower than 0.75. In these cases, a unique cut-off score was calculated for the *entire* sample adjusting by age and educational level.

The Digit Span Forward measure of WAIS-III (AUC = 0.69) did not meet the established AUC threshold. However, a valid global cut-off score was obtained, with good sensitivity and specificity values, after adjusting by age and education. Ideomotor praxis, Repetition and Verbal comprehension scores were not included in the analysis because of ceiling effects.

With regard to language of administration, although some statistically significant differences in test performances were found between individuals who were assessed in Spanish, and those who were examined in Catalan (see Table 14), most of general cut-offs obtained were similar in both languages (see Table 15). However, it was not possible to obtain cut-offs taking at the same time the age, education and language because that would be supposed an excessive reduction of the sample. Thus, after combining age by education by diagnostic and by language, there were 9 cells (the 37.5%) with, for example, less than 30 subjects.

**Table 14:** Comparison between Spanish and Catalan group performances on NBACE.

TEST	HS	MDS	F (3, 1526)	p
Global orientation	14.9 (0.5)	12.5 (2.0)	0.978	0.323
	*14.9 (0.4)*	*12.3 (2.0)*		
Verbal Learning WMS-III#	28.7 (5.8)	13.4 (4.8)	0.019	0.890
	*28.8 (5.7)*	*13.8 (4.7)*		
Delayed recall WMS-III	6.9 (2.4)	0.7 (1.3)	3.035	0.082
	*7.0 (2.5)*	*0.7 (1.3)*		
Forgetting WMS-III (%)	2.5 (1.7)	3.5 (1.5)	4.272	0.039
	*2.5 (1.6)*	*3.8 (1.6)*		
Recognition Memory WMS-III	22.5 (1.7)	16.8 (3.2)	0.048	0.827
	*22.7 (1.6)*	*17.0 (3.3)*		
Digit Span Forward WAIS-III	7.9 (1.9)	6.6 (1.8)	0.714	0.398
	*8.1 (1.8)*	*6.6 (1.6)*		
Digit Span Backwards WAIS-III	4.8 (1.7)	2.8 (1.4)	0.007	0.932
	*5.1 (1.6)*	*3.0 (1.5)*		
Block Design WAIS-III	3.8 (0.5)	2.3 (1.4)	14.035	<0.001*
	*3.9 (0.5)*	*2.1 (1.5)*		
Imitation praxis	3.8 (0.7)	2.2 (1.3)	10.427	0.001*
	*3.7 (0.8)*	*2.5 (1.2)*		
Global praxis	11.6 (0.9)	7.9 (2.3)	18.067	<0.001*
	*9.0 (2.6)*	*8.8 (2.3)*		
Visual Naming (15-BNT)	14.5 (0.9)	10.9 (3.2)	13.114	<0.001*
	*14.7 (0.6)*	*12.0 (2.7)*		
Poppelreuter’s test (responses)	9.8 (0.5)	7.9 (2.0)	6.359	0.012
	*9.9 (0.4)*	*8.4 (1.9)*		
15-OT (responses)‡	13.5 (1.6)	8.9 (2.7)	3.805	0.052
	*13.7 (1.6)*	*9.8 (2.4)*		
Luria’s Clock test	3.4 (0.7)	1.8 (1.2)	390.202	<0.001*
	*3.6 (0.7)*	*2.0 (1.3)*		
Automatic inhibition SKT (s)	2.5 (1.7)	3.5 (1.5)	4.272	0.039
	*2.5 (1.6)*	*3.8 (1.6)*		
Automatic Inhibition SKT (error)	1.0 (1.8)	7.5 (6.9)	1.935	0.164
	*0.7 (1.4)*	*6.4 (6.4)*		
Phonetic verbal fluency	13.8 (4.5)	7.3 (4.2)	0.033	0.856
	*14.2 (4.4)*	*7.8 (3.9)*		
Semantic verbal fluency	18.7 (4.8)	8.8 (3.8)	0.006	0.936
	*18.8 (4.7)*	*9.0 (3.8)*		
Similarities WAIS-III	11.34 (2.2)	6.8 (2.9)	1.942	0.164
	*11.5 (1.8)*	*7.3 (2.9)*		

HS: healthy subjects; MDS: mild dementia syndrome; WMS-III: Wechsler Memory Scale, Third Edition; WAIS-III: Wechsler Adult Intelligence Scale, Third edition; 15-BNT: the abbreviated Boston Naming Test with 15 items; 15-OT: The 15-Objects test; SKT: Syndrom Kurtz Test; s: time in seconds;

# Verbal learning WMS-III = 1^st^+2^nd^+3^rd^+4^th^ trial scores.

Spanish group values are written in regular print and Catalan group values in *italics*. Values are mean (standard deviation).

‡ In a subsample of 246 HS and 271 MDS.

* p<0.003 after Bonferroni’s correction.

**Table 15:** Cut-off scores in both languages (Spanish and Catalan).

	Spanish			Catalan		
TEST	Cut-off	SE	SP	Cut-off	SE	SP
Global orientation	<15	0.82	0.90	<15	0.84	0.89
Verbal learning WMS-III#	<21	0.92	0.92	<22	0.94	0.92
Delayed recall WMS-III	<4	0.95	0.94	<4	0.95	0.92
Forgetting WMS-III (%)	>47	0.90	0.86	>47	0.90	0.86
Recognition Memory WMS-III	<21	0.88	0.85	<22	0.89	0.81
Digit Span Forward WAIS-III	<4	0.76	0.99	<6	0.79	0.50
Digit Span Backwards WAIS-III	<4	0.84	0.62	<4	0.78	0.71
Block Design WAIS-III	<4	0.80	0.88	<4	0.69	0.90
Imitation praxis	<4	0.81	0.89	<4	0.70	0.88
Global praxis	<11	0.83	0.93	<12	0.86	0.81
Visual Naming (15-BNT)	<15	0.86	0.69	<15	0.80	0.80
Poppelreuter’s test (responses)	<10	0.78	0.85	<10	0.67	0.90
15-OT (responses)#	<13	0.90	0.77	<13	0.87	0.88
Luria’s Clock test	<3	0.69	0.91	<3	0.63	0.94
Automatic inhibition SKT (s)	>34	0.82	0.80	>31	0.79	0.81
Automatic Inhibition SKT (error)	>1	0.85	0.73	>1	0.79	0.80
Phonetic verbal fluency	<11	0.81	0.76	<11	0.78	0.80
Semantic verbal fluency	<14	0.89	0.86	<14	0.88	0.88
Similarities WAIS-III	<10	0.81	0.79	<10	0.76	0.89

SE: sensitivity; SP: specificity; WMS-III: Wechsler Memory Scale, Third Edition; WAIS-III: Wechsler Adult Intelligence Scale, Third edition; 15-BNT: the abbreviated Boston Naming Test with 15 items; 15-OT: The 15-Objects test; SKT: Syndrom Kurtz Test; s: time in seconds;

# Verbal learning WMS-III = 1^st^+2^nd^+3^rd^+4^th^ trial scores.

## Discussion

The present study reports the relevant diagnostic cut-off scores for a brief neuropsychological battery, the NBACE, for use in identifying subjects with cognitive impairment, especially dementia, in adults older than 44 years old. To our knowledge, this is the first study reporting sensitivity, specificity and AUC parameters of a neuropsychological battery for diagnosis of dementia in our country.

The AUC results demonstrated that most of NBACE cut-off scores were useful for detecting MDS in a sample of Spanish-Catalan speaking subjects, except for Repetition of sentences, Verbal Comprehension and Ideomotor praxis, which had ceiling effects in most of cases. Although Digit Span Forward subtest of WAIS-III showed a lower AUC value, the general cut-offs had good sensitivity and specificity values. The measures of verbal learning and long-term memory (including its recognition task) and Semantic Verbal Fluency were the most useful for detecting the presence of cognitive impairment or dementia. This is consistent with previous studies [47], [48], [49], and is probably due to the fact that most of MDS patients suffered AD.

As a limitation of the study, it has to be mentioned that participants were assessed in their mother tongue, but many of them spoke as well Catalan as Spanish. Then, it is uncertain about the percentage of subjects with balanced or unbalanced bilingualism (that is, with the ability to express high-proficiency equilibrate in both languages or not, respectively).

However, understanding the relevance of comparing performances and cut-offs between Spanish and Catalan groups, they have been reported. Non-verbal cognitive functions, such as visuospatial and visuoconstructive abilities showed statistically significant differences between groups, whereas for verbal cognitive functions, only visual naming showed differences between Catalan and Spanish speakers. Remarkably, in healthy and mild dementia groups, the Catalan group obtained better performances on visual naming than the Spanish one. Although further studies are needed to improve the knowledge about NBACE performances between bilingual and non-bilingual subjects, this finding is according with previous studies, reinforcing that bilingualism improves performance on visual naming in healthy subjects [50], but also in patients with dementia [51].

The results of the present study fit in with results from other previous studies, such as those from the Spanish Multicenter Normative Studies (NEURONORMA Project) [10]–[13], in an effort to provide norms for the neuropsychological assessment of cognitive functions in Spain. Moreover, as reported previously [19], the NBACE comprises an exhaustive clinically useful neuropsychological test battery that covers all the cognitive domains affected by neurodegenerative disorders. It can be administered in approximately 45 minutes, making it feasible to use in a clinical setting. The NBACE has been shown to be a useful tool able to detect cognitive impairment, especially dementia, in older than 44 years Spanish-speaking persons.

Data reported here are the next step of a previous study [19] in setting the relevant cut-off scores for impaired function, for different levels of age and education, and decision making algorithms for use in identifying adult individuals with cognitive impairment. *Fundació ACE* enrolls more than 1000 new patients every year, all of whom are assessed by the NBACE.

In the present study, the neuropsychological assessment was satisfied in a Diagnostic Unit with specific demands and needs. This is an intrinsic limitation for future replication processes when other disorders or severities are considered. To validate these cut-off scores in other clinical samples, replication studies would be useful. As a next step, further studies are needed to assess the usefulness of NBACE tests battery in discriminating among different types of dementia.

## References

[1] Pasquier F (1999) Early diagnosis of dementia: Neuropsychology. J Neurol 246: 6–15. 9987708 10.1007/s004150050299

[2] Maruta C, Guerreiro M, de Mendonça A, Hort J, Scheltens P (2011) The use of neuropsychological tests across Europe: the need for a consensus in the use of assessment tools for dementia. Eur J Neurol 18: 279–285. 20597968 10.1111/j.1468-1331.2010.03134.x

[3] Nordlund A, Rolstad S, Hellström P, Sjögren M, Hansen S, et al (2005) The Goteborg MCI study: mild cognitive impairment is a heterogeneous condition. J Neurol Neurosurg Psychiatry 76: 1485–1490. 16227535 10.1136/jnnp.2004.050385 PMC1739388

[4] Artiola L, Hermosillo D, Heaton R, Pardee RE (1999) Manual de normas y procedimientos para la batería neuropsicológica en español. Arizona: M. Press Tucson.

[5] Benton AL, Hamsher KS, Varney NR, Spreen O (1983) Contributions to Neuropsychological Assessment: A Clinical Manual. New York: Oxford University Press.

[6] Heaton RK, Taylor MJ (2004) Revised Comprehensive Norms for an Expanded Halstead-Reitan battery: Demographically Adjusted Neuropsychological Norms for African, American and Caucasian Adults. Odessa, FL: Psychological Assessment Resources.

[7] Mitroshima M, Boone KB, Razani J, D’Elia LF (2005) Handbook of Normative Data for Neuropsychological Assessment. London: Oxford University Press.

[8] Chester JG, Grande LG, Milberg WP, McGlinchey RE, Lipsitz LA, et al (2011) Cognitive screening in community-dwelling elders: performance on the clock-in-the-box. Am J Med 124: 662–669. 21592451 10.1016/j.amjmed.2011.02.023 PMC3128995

[9] Acevedo A, Krueger KR, Navarro E, Ortiz F, Manly JJ, et al (2009) The Spanish translation and adaptation of the Uniform Data Set of the National Institute on Aging Alzheimer’s Disease Centers. Alzheimer Dis Assoc Disord 23: 102–109. 19474568 10.1097/WAD.0b013e318193e376 PMC2963171

[10] Peña-Casanova J, Quintana-Aparicio M, Quiñones-Ubeda S, Aguilar M, Molinuevo JL, et al (2009) Spanish Multicenter normative Studies (NEURONORMA Project): norms for the visual object and space perception battery-abbreviated, and judgment of line orientation. Arch Clin Neuropsychol 24: 355–370. 19648584 10.1093/arclin/acp040

[11] Peña-Casanova J, Gramunt-Fombuena N, Quiñones-Ubeda S, Sánchez-Benavides G, Aguilar M, et al (2009) Spanish Multicenter Normative Studies (NEURONORMA Project): norms for the Rey-Osterrieth complex figure (copy and memory), and free and cued selective reminding test. Arch Clin Neuropsychol 24: 371–393. 19661107 10.1093/arclin/acp041

[12] Peña-Casanova J, Quiñones-Ubeda S, Gramunt-Fombuena N, Aguilar M, Casas L, et al (2009) Spanish Multicenter normative Studies (NEURONORMA Project): Norms for Boston Naming Test and Token Test. Arch Clin Neuropsychol 24: 343–354. 19648582 10.1093/arclin/acp039

[13] Peña-Casanova J, Quiñones-Ubeda S, Gramunt-Fombuena N, Quintana-Aparicio M, Aguilar M, et al (2009) Spanish Multicenter normative Studies (NEURONORMA Project): Norms for verbal fluency tests. Arch Clin Neuropsychol 24: 395–411. 19648583 10.1093/arclin/acp042

[14] Rami L, Serradell M, Bosch B, Caprile C, Sekler A, et al (2008) Normative data for the Boston Naming Test and the Piramids and Palm Trees Test in the elderly Spanish population. J Clin Exp Neuropsychol 30: 1–6. 18165935 10.1080/13803390701743954

[15] Peña-Casanova J, Aguilar M, Santacruz P, Bertran-Serra I, Hernández G, et al (1997) [Adaptation and normalization of the Alzheimer’s disease Assessment Scale for Spain (NORMACODEM) (II)]. Neurología 12: 69–77. 9147454

[16] Peña-Casanova J, Guardia J, Bertran-Serra I, Manero RM, Jarne A (1997) [Versión abreviada del test Barcelona (I): subtests y perfiles normales]. Neurología 12: 99–111. 9198458

[17] Quintana M, Peña-Casanova J, Sánchez-Benavides G, Langohr K, Manero RM, et al (2011) Spanish multicenter normative studies (Neuronorma project): norms for the abbreviated Barcelona Test. Arch Clin Neuropsychol 26: 144–157. 21149392 10.1093/arclin/acq098

[18] Manubens JM, Barandiarán M, Martínez-Lage P, Francés I, Martínez C, et al (2005) [Values of GERMCIDE neuropsychological protocol in a sample of normal subjects]. Neurología 20: 174–179. 15891946

[19] Alegret M, Espinosa A, Vinyes-Junqué G, Valero S, Hernández I, et al (2012) Normative data of a brief neuropsychological battery for Spanish individuals older than 49. J Clin Exp Neuropsychol 34: 209–219. 22149440 10.1080/13803395.2011.630652 PMC3264764

[20] Petersen RC (2004) Mild cognitive impairment as a diagnostic entitiy. J Intern Med 256: 183–194. 15324362 10.1111/j.1365-2796.2004.01388.x

[21] Lezak MD, Howieson DB, Bigler ED, Tranel D (2012) Neuropsychological Assessment, Fifth Edition. New York: Oxford University Press.

[22] Pendlebury ST, Mariz J, Bull L, Mehta Z, Rothwell PM (2012) MoCA, ACE-R, and MMSE versus the National Institute of Neurological Disorders and Stroke-Canadian Stroke Network Vascular Cognitive Impairment Harmonization Standards Neuropsychological Battery after TIA and stroke. Stroke 43: 464–469. 22156700 10.1161/STROKEAHA.111.633586 PMC5390857

[23] Nasreddine ZS, Phillips NA, Bédirian V, Charbonneau S, Whitehead V, et al (2005) The Montreal Cognitive Assessment, MoCA: a brief screening tool for mild cognitive impairment. J Am Geriatr Soc 53: 695–699. 15817019 10.1111/j.1532-5415.2005.53221.x

[24] Freitas S, Simões MR, Alves L, Santana I (2013) Montreal Cognitive Assessment: Validation Study for Mild Cognitive Impairment and Alzheimer Disease. Alzheimer Dis Assoc Disord 27: 37–43. 22193353 10.1097/WAD.0b013e3182420bfe

[25] Hänninen T, Pulliainen V, Sotaniemi M, Hokkanen L, Salo J, et al (2010) [Early detection of cognitive changes in memory diseases: new cut-off scores for the Finnish version of CERAD neuropsychological battery]. Duodecim 126: 2013–2021. 21053518

[26] Sylvestre G, Chopard G, Tio G, Magnin E, Rumbach L, et al (2011) [Diagnostic norms of RAPID neuropsychological battery tests for subjects aged 60 to 89 years with Alzheimer’s disease]. Rev Neurol (Paris) 167: 495–504. 21474155 10.1016/j.neurol.2010.12.004

[27] Blesa R, Pujol M, Aguilar M, Santacruz P, Bertran-Serra I, et al (2001) Clinical validity of the “mini-mental state” for Spanish speaking communities. Neuropsychologia 39: 1150–1157. 11527552 10.1016/s0028-3932(01)00055-0

[28] Del Ser Quijano T, Sánchez F, García de Yébenes MJ, Otero A, Zunzunegui MV, et al (2004) [Spanish version of the 7 Minute screening neurocognitive battery. Normative data of an elderly population sample over 70]. Neurología 19: 344–358. 15273881

[29] Böhm P, Peña-Casanova J, Gramunt N, Manero RM, Terrón C, et al (2005) [Spanish version of the Memory Impairment Screen (MIS): normative data and discriminant validity]. Neurología 20: 402–411. 16217689

[30] Rami L, Bosch B, Valls-Pedret C, Caprile C, Sánchez-Valle Díaz R, et al (2009) [Discriminatory validity and association of the mini-mental test (MMSE) and the memory alteration test (M@T) with a neuropsychological battery in patients with amnestic mild cognitive impairment and Alzheimer’s disease]. Rev Neurol. 49: 169–174. 19621317

[31] Boada M, Tárraga L, Hernández I, Valero S, Alegret M, et al.. (2013) Design of a Comprehensive Alzheimer’s Disease Clinic and Research Center in Spain to Meet Critical Patient and Family Needs. Alzheimers Dement (In Press). 10.1016/j.jalz.2013.03.006 PMC3951687 24035148

[32] Morris JC (1993) The Clinical Dementia Rating (CDR): Current version and scoring rules. Neurology 43: 2412–2414. 10.1212/wnl.43.11.2412-a 8232972

[33] Solomon PR, Pendlebury WW (1998) Recognition of Alzheimer’s disease: the 7 Minute Screen. Fam Med 30: 265–271. 9568495

[34] Del Ser T, García de Yébenes MJ, Sánchez F, Frades B, Rodríguez A, et al (2004) [Cognitive assessment in the elderly. Normative data of a Spanish population sample older than 70 years]. Med Clin (Barc) 122: 727–740. 15171906 10.1157/13062190

[35] Blessed G, Tomlinson BE, Roth M (1968) The association between quantitative measures of dementia and of senile change in the cerebral grey matter of elderly subjects. Br J Psychiatry 114: 797–811. 5662937 10.1192/bjp.114.512.797

[36] Peña-Casanova J, Aguilar M, Bertran-Serra I, Santacruz P, Hernández G, et al (1997) [Normalization of cognitive and functional assessment instruments for dementia (NORMACODEM) (I): objectives, content and population]. Neurología 12: 61–8. 9147453

[37] Linn MW, Linn BS (1982) The Rapid Disability Rating Scale-2. J Am Geriatr Soc 30: 378–82. 7077018 10.1111/j.1532-5415.1982.tb02835.x

[38] Tinetti ME, Williams TF, Mayewski R (1986) Fall risk index for elderly patients based 524 on number of chronic disabilities. Am J Med 80: 429–434. 3953620 10.1016/0002-9343(86)90717-5

[39] Hachinski VC, Iliff LD, Zilhka E, Du Boulay GH, McAllister VL, et al (1975) Cerebral blood flow in dementia. Arch Neurol 32: 632–637. 1164215 10.1001/archneur.1975.00490510088009

[40] Boada M, Cejudo JC, Tárraga L, López OL, Kaufer D (2002) [Neuropsychiatric inventory questionnaire (NPI-Q): Spanish validation of an abridged form of the Neuropsychiatric Inventory (NPI)]. Neurología 17: 317–323. 12084358

[41] McKhann GM, Knopman DS, Chertkow H, Hyman BT, Jack CR Jr, et al (2011) The diagnosis of dementia due to Alzheimer’s disease: recommendations from the National Institute on Aging-Alzheimer’s Association workgroups on diagnostic guidelines for Alzheimer’s disease. Alzheimers Dement 7: 263–269. 21514250 10.1016/j.jalz.2011.03.005 PMC3312024

[42] Román GC, Tatemichi TK, Erkinjuntti T, Cummings JL, Masdeu JC, et al (1993) Vascular dementia: diagnostic criteria for research studies. Report of the NINDS-AIREN International Workshop. Neurology 43: 250–260. 8094895 10.1212/wnl.43.2.250

[43] Neary D, Snowden JS, Gustafson L, Passant U, Stuss D, et al (1998) Frontotemporal lobar degeneration: a consensus on clinical diagnostic criteria. Neurology 51: 1546–1554. 9855500 10.1212/wnl.51.6.1546

[44] Rascovsky K, Hodges JR, Knopman D, Mendez MF, Kramer JH, et al (2011) Sensitivity of revised diagnostic criteria for the behavioural variant of frontotemporal dementia. Brain 134: 2456–2477. 21810890 10.1093/brain/awr179 PMC3170532

[45] Poewe W, Gauthier S, Aarsland D, Leverenz JB, Barone P, et al (2008) Diagnosis and management of Parkinson’s disease dementia. Int J Clin Pract 62: 1581–1587. 18822028 10.1111/j.1742-1241.2008.01869.x PMC2658001

[46] McKeith IG, Dickson DW, Lowe J, Emre M, O’Brien JT, et al (2005) Diagnosis and management of dementia with Lewy bodies: third report of the DLB Consortium. Neurology 65: 1654–1656. 16237129 10.1212/01.wnl.0000187889.17253.b1

[47] Rountree SD, Waring SC, Chan WC, Lupo PJ, Darby EJ, et al (2007) Importance of subtle amnestic and nonamnestic deficits in mild cognitive impairment: Prognosis and conversion to dementia. Dement Geriatr Cogn Disord 24: 476–482. 17992015 10.1159/000110800

[48] Dubois B, Feldman HH, Jacova C, Dekosky ST, Barberger-Gateau P, et al (2007) Research criteria for the diagnosis of Alzheimer’s disease: Revising the NINCDS-ADRDA criteria. Lancet Neurol 6: 734–746. 17616482 10.1016/S1474-4422(07)70178-3

[49] Espinosa A, Alegret M, Valero S, Vinyes-Junqué G, Hernández I, et al (2013) A Longitudinal Follow-Up of 550 Mild Cognitive Impairment Patients: Evidence for Large Conversion to Dementia Rates and Detection of Major Risk Factors Involved. J Alzheimers Dis 34: 769–780. 23271318 10.3233/JAD-122002

[50] Gollan TH, Fennema-Notestine C, Montoya RI, Jernigan TL (2007) The bilingual effect on Boston Naming Test performance. J Int Neuropsychol Soc. 2007 Mar 13(2): 197–208. 10.1017/S1355617707070038 17286875

[51] Gollan TH, Salmon DP, Montoya RI, da Pena E (2010) Accessibility of the nondominant language in picture naming: a counterintuitive effect of dementia on bilingual language production. Neuropsychologia 48: 1356–66. 20036679 10.1016/j.neuropsychologia.2009.12.038 PMC2843816

