# Correction: Cut-off Scores of a Brief Neuropsychological Battery (NBACE) for Spanish Individual Adults Older than 44 Years Old

**DOI:** 10.1371/annotation/acefbfc7-8fb6-4505-a273-0fdb4e2bb580

**Published:** 2014-01-15

**Authors:** Montserrat Alegret, Ana Espinosa, Sergi Valero, Georgina Vinyes-Junqué, Agustín Ruiz, Isabel Hernández, Maitee Rosende-Roca, Ana Mauleón, James T. Becker, Lluís Tárraga, Mercè Boada

There are errors in Tables 8 and 12 of this article. In Table 8, the range is incorrect. The correct range is 0-15. In Table 12, the range is incorrect. The correct range is >0.

